# Census of breeding birds in the Pyrenees in the early 1980s: A publicly available dataset for ecological research

**DOI:** 10.1016/j.dib.2023.109619

**Published:** 2023-09-24

**Authors:** Michel Génard, Françoise Lescourret

**Affiliations:** INRAE, PSH, 228 route de l'Aérodrome, 84914 Avignon, France

**Keywords:** Bird, Mountain, Point-count, Pine forest, Heath, Grassland, Cropland

## Abstract

The Pyrenees is a mountain range in south-western Europe that supports a rich diversity of bird species. In 1981, point count surveys of breeding birds (passerines and picidae) were carried out in the Vanera valley, a valley in the eastern Pyrenees, resulting in a data set of 228 counts. These data provide valuable information on the distribution and abundance of bird populations on 5350 ha of heterogeneous land (including cropland, grasslands, heaths and river banks, and pine forest) at altitudes ranging from 1100 to 2600 meters. Additional point count surveys were carried out in the pine forest from 1982 to 1985, resulting in a data set of 144 counts. Habitat descriptors (percentage of herbaceous and ligneous plant cover at different heights - 0-1m, 1-4m and more than 8m; vegetation type) and altitude were assessed around each point count.

This dataset provides a complete picture of the breeding bird community in a typical valley of eastern Pyrenees in the early 1980s, which could be compared with future censuses to contribute to a variety of research questions, such as quantifying changes in birds between the early 1980s and now in mountain areas, understanding the effects of climate change on bird populations, examining the effects of habitat fragmentation and land-use change, and identifying priority areas for conservation and management. These data could inspire new research and contribute to our collective understanding of bird ecology in the Pyrenees.

Specifications Table

**Table:** 

Subject	Biology
Specific subject area	Biodiversity
Type of data	Table
How the data were acquired	Bird point counts with unlimited distance. Each count lasts 20 mn. Within 50 m of each point count, altitude and the type of vegetation were recorded and the percentage cover of different vegetation strata were estimated by comparison with reference drawings representing imaginary cover levels of 5%, 10%, etc.
Data format	Raw
Description of data collection	One year of sampling in a mountain valley characterised by 9 vegetation types. 5 years of sampling in the pine forest (30% of the study area). Birds were counted in the morning in spring and habitat descriptors were measured after each point count.
Data source location	INRAE Vanera valley, Eastern Pyrenees France 42° 23’ 14’’N 2° 2’ 7’’E (Latitude: 42.3872; Longitude: 2.0352) for the village of Valcebollère (Fig. 1)
Data accessibility	Repository name: Data INRAE Data identification number: doi:10.57745/YUESL8 Direct URL to data: https://entrepot.recherche.data.gouv.fr/dataset.xhtml?persistentId=doi:10.57745/YUESL8
Related research article	M. Génard, F. Lescourret, Combining Niche and Dispersal in a Simple Model (NDM) of Species Distribution, PLoS ONE. 8 (2013) e79948. https://doi.org/10.1371/journal.pone.0079948

## 1 Value of the Data

• These data give a picture of the bird communities in the Pyrenees in the early 1980s. They are useful for differentiating bird communities in different habitats and according to altitude. They are also useful for analyzing the stability of pine communities over 5 years.
• In addition to birds, these data indicate the altitude, vegetation type and vegetation cover at different heights, which characterize the habitats surveyed
• These data can be useful for researchers working on bird communities and biodiversity, but also on typical mountain species such as the Common Crossbill.
• These data should be of interest to researchers concerned with the evolution of biodiversity or the abundance of species of interest since the 1980s in Mediterranean mountain areas. Each point count is positioned on a map and new counts could easily be carried out at the same locations.
• These data should be useful for biogeographical comparisons of bird communities with other mountain or lowland sites.

## 2 Objective

The surveys carried out give a good picture of the bird communities living in the different environments of a valley in the eastern Pyrenees, the Vanera valley, in the 1980s. The climate was colder then, and the region has experienced frequent heat waves and droughts in recent decades. The land cover is also slightly different today from what it was in the 1980s, due to changes in human activities. Our data could be used as a reference (possibly together with others collected at other sites in the Pyrenees in the same years) for studies on the evolution of bird communities as a result of global changes that have taken place in recent decades.

## 3 Data Description

**Fig. 1:**
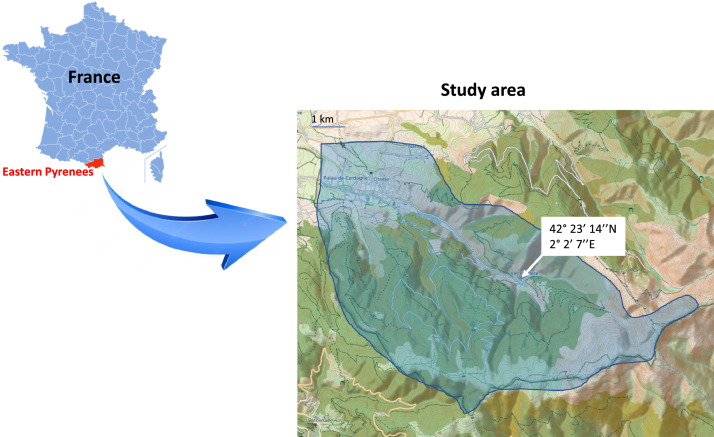
Location of the Eastern Pyrenees and the study area in blue on the topographic map provided by openstreet map. Forested areas are shown in green.

The data presented here consist of records of breeding birds from fields, grasslands, heaths and forests at altitudes between 1100 and 2600 m in the Vanera valley, a typical valley in the eastern Pyrenees (Fig. 1). A total of 60 species and 5305 individuals were recorded (Table 1).

**Table 1:** Total number of individuals recorded per bird species per year during point counts in the Vanera valley, Eastern Pyrenees.

Bird species	1981	1982	1983	1984	1985	Sum
Parus major	79	0	0	0	0	79
Cyanistes caeruleus	15	0	0	0	0	15
Periparus ater	421	210	52	52	59	794
Lophophanes cristatus	87	63	11	16	16	193
Sitta europaea	6	0	0	0	0	6
Certhia brachydactyla	47	32	5	13	11	108
Troglodytes troglodytes	59	28	6	12	9	114
Turdus viscivorus	11	21	2	4	1	39
Turdus philomelos	38	29	9	6	21	103
Turdus merula	152	4	0	0	2	158
Saxicola rubicola SRB	18	0	0	0	0	18
Luscinia megarhynchos	63	0	0	0	0	63
Erithacus rubecula	140	116	18	27	17	318
Sylvia atricapilla	92	0	0	0	0	92
Sylvia borin	57	4	0	0	0	61
Sylvia communis	50	1	0	0	0	51
Phylloscopus collybita	12	5	0	0	0	17
Phylloscopus bonelli	34	0	0	0	0	34
Regulus regulus	85	70	22	29	24	230
Prunella modularis	207	41	8	9	9	274
Anthus trivialis	67	20	1	5	1	94
Carduelis carduelis	11	0	0	0	0	11
Carduelis cannabina	21	0	0	0	0	21
Serinus serinus	34	3	0	0	0	37
Pyrrhula pyrrhula	32	24	1	2	0	59
Fringilla coelebs	294	122	46	61	29	552
Emberiza citrinella	31	1	0	0	0	32
Alauda arvensis	296	17	2	1	3	319
Turdus torquatus	20	12	1	3	5	41
Saxicola rubetra	14	0	0	0	0	14
Serinus citrinella	243	74	53	34	29	433
Loxia curvirostra	138	162	38	43	22	403
Emberiza hortulana	5	0	0	0	0	5
Emberiza cia	43	0	0	0	0	43
Phoenicurus ochruros	25	6	1	4	5	41
Lullula arborea	31	4	0	1	1	37
Anthus spinoletta	30	4	0	0	0	34
Emberiza calandra	15	0	0	0	0	15
Oenanthe oenanthe	29	0	0	0	0	29
Prunella collaris	11	0	0	0	0	11
Aegithalos caudatus	1	0	0	0	0	1
Sturnus vulgaris	5	0	0	0	0	5
Emberiza cirlus	3	0	0	0	0	3
Certhia familiaris	3	0	0	0	0	3
Spinus spinus	3	0	0	2	0	5
Dendrocopos major	32	29	9	13	9	92
Oriolus oriolus	5	0	0	0	0	5
Garrulus glandarius	8	1	0	1	0	10
Monticola saxatilis	2	0	0	0	0	2
Hypolais polyglotta	1	0	0	0	0	1
Jynx torquilla	6	0	0	0	0	6
Motacilla cinerea	4	0	0	0	0	4
Lanius senator	1	0	0	0	0	1
Lanius collurio	2	0	0	0	0	2
Petronia petronia	10	0	0	0	0	10
Picus viridis	23	0	0	0	0	23
Upupa epops	10	0	0	0	0	10
Coturnix coturnix	9	0	0	0	0	9
Columba palumbus	21	7	6	2	0	36
Cuculus canorus	78	0	1	0	0	79
**Sum**	3290	1110	292	340	273	5305

**Fig. 2:**
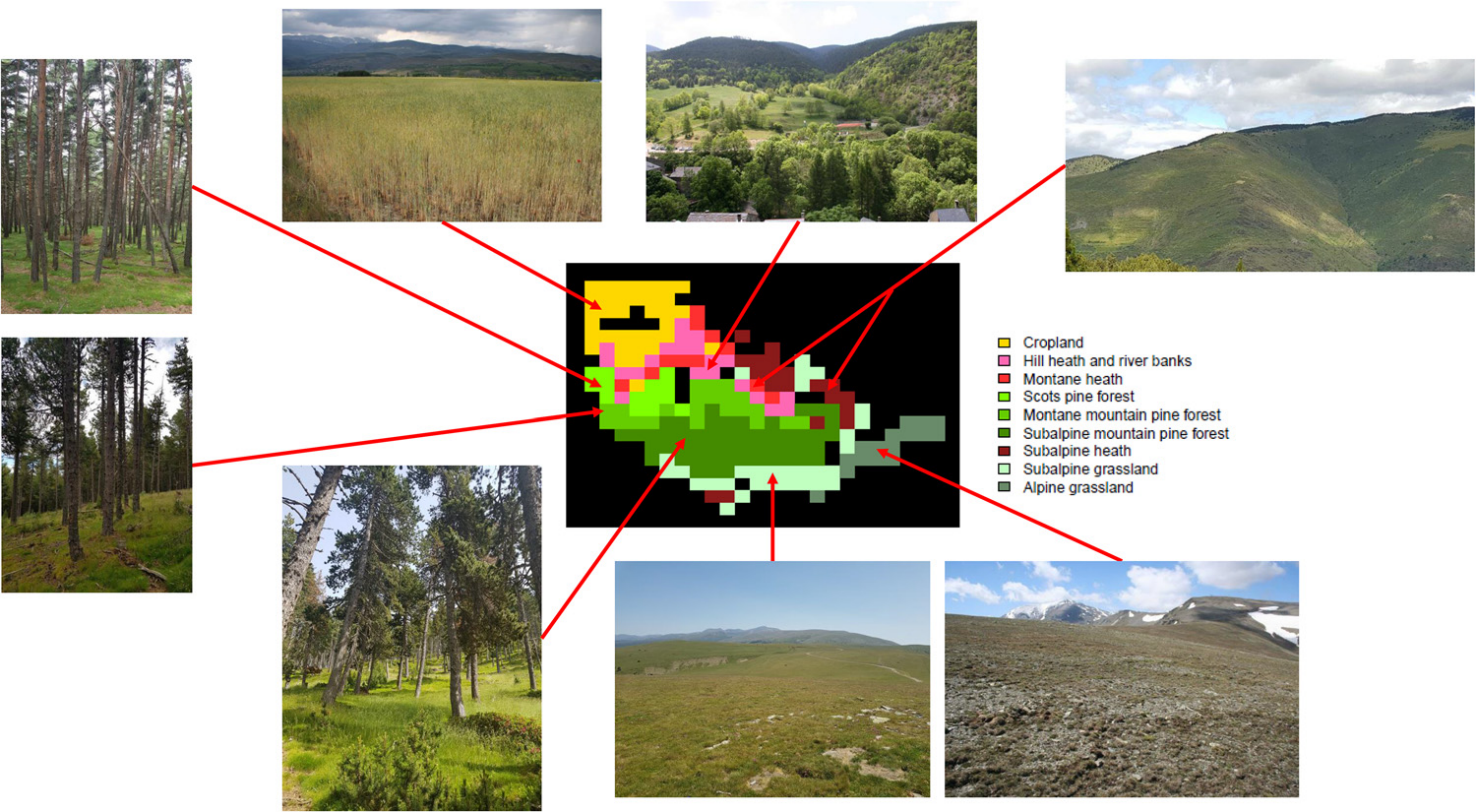
Vegetation types found in the study area.

In 1981, 228 bird counts were carried out on 5350 ha of heterogeneous land (Fig. 2). In 1982, 1983, 1984 and 1985, 75, 22, 26 and 21 bird counts respectively were made only in the pine forest (30% of the study area). The percentage of herbaceous and ligneous plant cover at different heights (0–1 m, 1–4 m and more than 8 m) was assessed within a 50 m radius of the bird count site. In addition, altitude was recorded and nine vegetation types were defined: cropland, hill heath and river banks, montane heath, Scots pine (Pinus sylvestris L.) forest, montane mountain pine (Pinus uncinata Ram) forest, subalpine mountain pine forest, subalpine heath, subalpine grassland, and alpine grassland (Fig. 2).

## 4 Experimental Design, Materials and Methods

Birds were sampled using the point-count technique with unlimited distance [1]. Singing passerines and *picidae* were recorded for 20 min in spring mornings. The bird species were identified by expertise ornithologist (F.L. and M.G.).

**Fig. 3:**
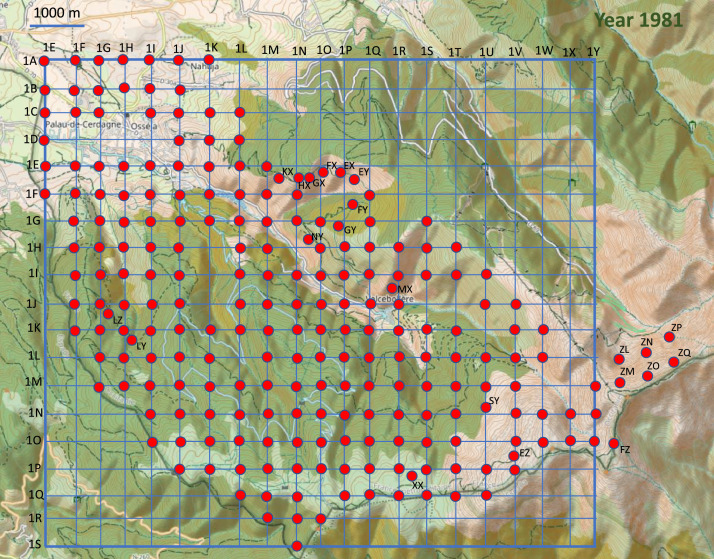
Location of the 228 point counts surveyed in 1981 on the topographic map of the Vanera valley. The name of the grid points is 1XY (X=abscissa and Y= ordinate). For example, the first point on the top left of the grid is 1EA. The labels of the points outside the grid are shown on the map.

**Fig. 4:**
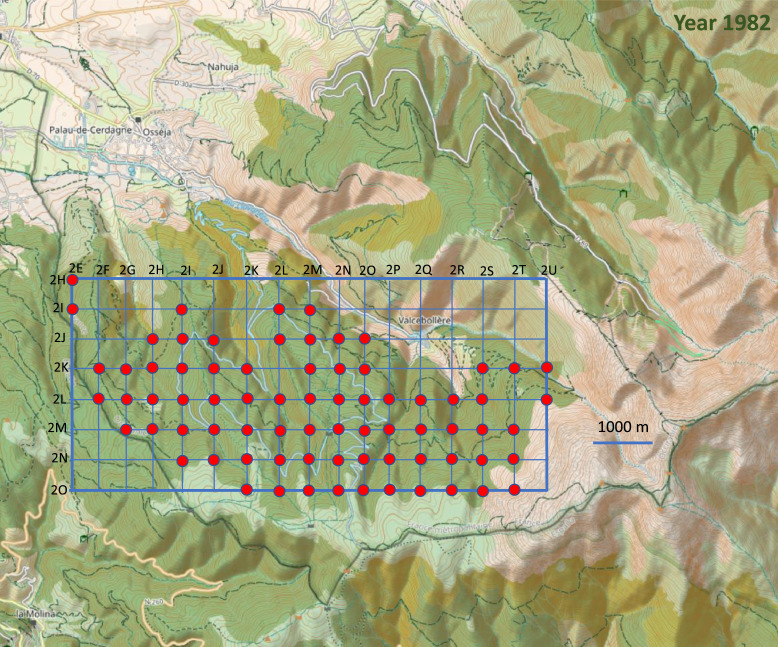
Location of the 75 point counts surveyed in 1982 on the topographic map of the Vanera valley. The name of the grid points is 1XY (X=abscissa and Y= ordinate). For example, the first point on the top left of the grid is 2EH.

**Fig. 5:**
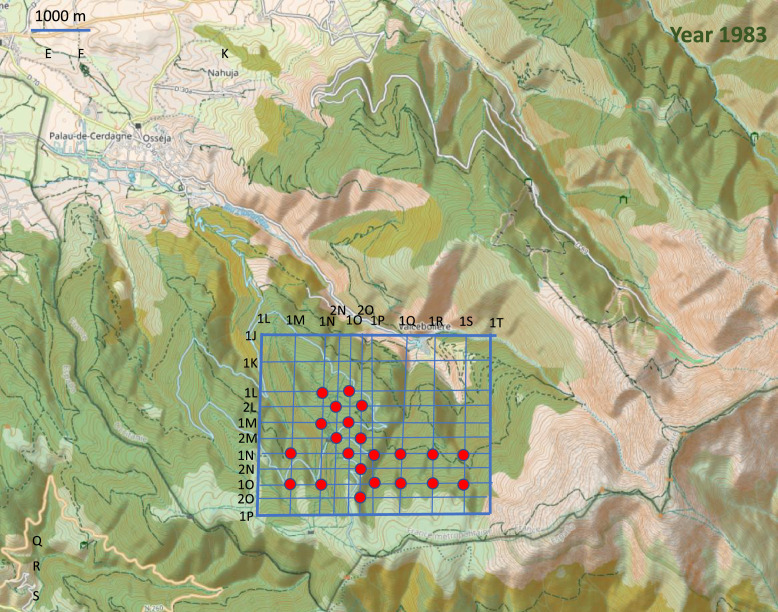
Location of the 22 point counts surveyed in 1983 on the topographic map of the Vanera valley. The name of the grid points is 1XY (X=abscissa and Y= ordinate). For example, the first point on the top left of the grid is 1NL.

**Fig. 6:**
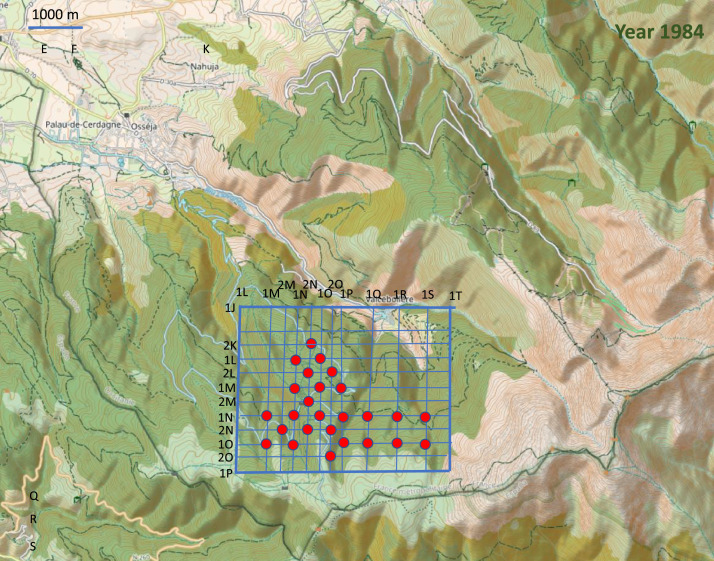
Location of the 26 point counts surveyed in 1984 on the topographic map of the Vanera valley. The name of the grid points is 1XY (X=abscissa and Y= ordinate). For example, the point on the top of the grid is 2NK.

**Fig. 7:**
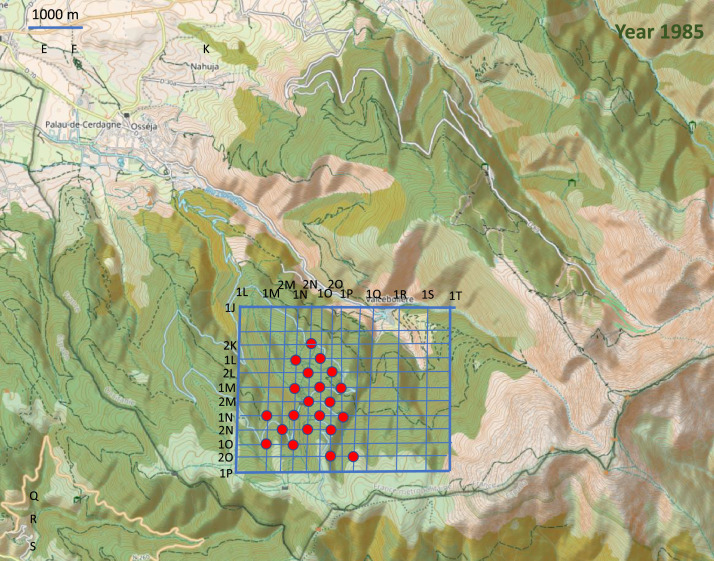
Location of the 21 point counts surveyed in 1985 on the topographic map of the Vanera valley. The name of the grid points is 1XY (X=abscissa and Y= ordinate). For example, the first point on the top of the grid is 2NK.

Point-counts were conducted in the centre of 0.025 km² square cells in fair weather (no wind or rain). The cells were mainly situated on a grid that covered the study area (Fig. 3, Fig. 4, Fig. 5, Fig. 6, Fig. 7). Habitat descriptors were evaluated within a 50 m radius of the point-count site. Plants cover were estimated through comparison with reference drawings depicting imaginary cover levels of 5%, 10%, and so on [2]. The altitude was measured using an altimeter and the IGN map at 1:50,000 scale.

## Ethics Statements

This work meets the ethical requirements for publication. It did not involve human subjects, laboratory animals or disturbance of wild animals.

## CRediT authorship contribution statement

**Michel Génard:** Data curation, Writing – original draft. **Françoise Lescourret:** Data curation, Writing – original draft.

## Data Availability

Census of breeding birds in a typical valley of Eastern Pyrenees (Original data) (Recherche Data Gouv).
